# The Impact of User Engagement With Exposure Components on Posttraumatic Stress Symptoms in an mHealth Mobile App: Secondary Analysis of a Randomized Controlled Trial

**DOI:** 10.2196/49393

**Published:** 2024-07-18

**Authors:** C Adrian Davis, Madeleine Miller, Carmen P McLean

**Affiliations:** 1 National Center for Posttraumatic Stress Disorder, Dissemination and Training Division, VA Palo Alto Healthcare System, Menlo Park, CA, United States; 2 Health Psychology and Clinical Science, The Graduate Center, The City University of New York, New York, NY, United States; 3 Department of Psychiatry and Behavioral Sciences, Stanford University, Stanford, CA, United States

**Keywords:** posttraumatic stress disorder, PTSD, mHealth apps, user engagement, mHealth interventions, digital interventions, veterans

## Abstract

**Background:**

Mobile mental health apps are a cost-effective option for managing mental health problems, such as posttraumatic stress disorder (PTSD). The efficacy of mobile health (mHealth) apps depends on engagement with the app, but few studies have examined how users engage with different features of mHealth apps for PTSD.

**Objective:**

This study aims to examine the relationship between app engagement indices and PTSD symptom reduction using data from an unblinded pilot randomized controlled trial of “Renew” (Vertical Design), an exposure-based app for PTSD with and without coaching support. Because exposure is an effective approach for treating PTSD, we expected that engagement with exposure activities would be positively related to symptom reduction, over and above overall app usage.

**Methods:**

Participants were veterans (N=69) with clinically significant PTSD symptoms who were recruited online using Facebook advertisements and invited to use the Renew app as often as they wanted over a 6-week period. Participants completed screening and assessments online but provided informed consent, toured the app, and completed feedback interviews via telephone. We assessed users’ self-reported PTSD symptoms before and after a 6-week intervention period and collected app usage data using a research-instrumented dashboard. To examine overall app engagement, we used data on the total time spent in the app, the number of log-in days, and the number of points that the user gained in the app. To examine engagement with exposure components, we used data on total time spent completing exposure activities (both in vivo and imaginal), the number of in vivo exposure activities completed, and the number of characters written in response to imaginal exposure prompts. We used hierarchical regression analyses to test the effect of engagement indices on change in PTSD symptoms.

**Results:**

Usage varied widely. Participants spent an average of 166.09 (SD 156.52) minutes using Renew, over an average of 14.7 (SD 10.71) mean log-in days. Engagement with the exposure components of the app was positively associated with PTSD symptom reduction (*F*_6,62_=2.31; *P*=.04). Moreover, this relationship remained significant when controlling for overall engagement with the app (Δ*F*_3,62_=4.42; *P*=.007). The number of characters written during imaginal exposure (*β*=.37; *P*=.009) and the amount of time spent completing exposure activities (*β*=.36; *P*=.03) were significant contributors to the model.

**Conclusions:**

To our knowledge, this is the first study to show a relationship between symptom improvement and engagement with the active therapeutic components of an mHealth app (ie, exposure) for PTSD. This relationship held when controlling for overall app use, which suggests that it was engagement with exposure, specifically, that was associated with symptom change. Future work to identify ways of promoting greater engagement with self-guided exposure may help improve the effectiveness of mHealth apps for PTSD.

## Introduction

### Overview

Mobile mental health apps have continued to proliferate in recent years [1]. As of last year, more than 80 mobile mental health (mHealth) apps aimed at managing posttraumatic stress disorder (PTSD)—a significant public health concern with an estimated 7% lifetime prevalence in the United States of America—are now available for download [2,3]. Research on mHealth apps for PTSD has also increased and suggests that these tools are generally feasible and acceptable to users [4]. However, a recent review found that evidence for the efficacy of PTSD mHealth apps was not consistently superior to inactive control conditions [5].

The efficacy of an mHealth app for PTSD will depend upon how the user engages with the tool. Only one study has examined the relationship between engagement with a PTSD self-management app and symptom change. This study found that the number of log-ins to the mHealth app PTSD Coach (Vertical Design) was not related to PTSD symptom reduction [6]. This contrasts with findings from depression mHealth apps showing that number of log-ins [7] and number of activities per log-in [8] are linked with change in depressive symptoms. Additional research on the relationship between app engagement and PTSD symptom change is clearly needed and could inform user guidance on how to benefit from using PTSD mHealth self-management apps.

### Objectives

The current analysis aims to address this gap by exploring the relationship between user engagement indices and symptom reduction in users of a PTSD mHealth self-management app called “Renew” [9,10]. Based on principles of exposure therapy, Renew uses gamified elements and activities designed to help users target their PTSD symptoms using in vivo and written exposure. Given the evidence supporting exposure as an efficacious therapeutic approach for treating PTSD [11], we hypothesized that engagement with the exposure activities in Renew would be uniquely related to symptom reduction, over and above general app use.

## Methods

### Participants

Potential participants were recruited through Facebook (Meta) advertisements because we were interested in examining a nationally representative sample of veterans. As of the Veterans Health Administration’s last publicly released report, more than half of veterans do not receive care through the Department of Veterans Affairs (VA) [12], so a sample recruited strictly from among VA patients would not necessarily represent veterans as a whole. Potential participants completed an online screening measure through REDCap (Research Electronic Data Capture; Vanderbilt University) [13] to assess veteran status, trauma exposure [14], and PTSD symptoms. Eligible participants were veterans with clinically significant PTSD symptoms, defined by a score of ≥31 on the Posttraumatic Stress Disorder Checklist for *DSM-5* (PCL-5) [14]; access to an Android phone (Renew was only developed for Android OS); and sufficient smartphone literacy and internet connection to successfully download the app with telephone guidance from study staff. There were no exclusion criteria.

### Ethical Considerations

All participants gave their informed consent for inclusion before they participated in this trial. The protocol was approved by the Stanford University Institutional Review Board (52829).

### Renew

Renew is a research-instrumented mobile app designed to reduce symptoms of PTSD using in vivo exposure and imaginal exposure, developed by Vertical Design. Other components include psychoeducation, behavioral activation through self-care, and assessment. Users gain points for completing activities in the app. Renew was designed to be used both with and without human coaching support, provided through a one-way messaging function. Users downloaded Renew from the Google Play Store and accessed the intervention using an invitation code provided by the study team during app orientation. Readers of this article who possess an Android phone may email the corresponding author to receive a Renew invitation code if they wish to access the intervention. Before this trial, we tested the feasibility and acceptability of Renew in a small pilot study, after which the content of Renew was updated according to user feedback and frozen throughout the duration of the current trial. Miller et al [10] and McLean et al [9] give more details about Renew, including the details of the feasibility and acceptability pilot study.

### Measures

#### Renew Usage Data

Participant usage data were collected through a secure research dashboard. User access to the app was restricted to only those users who were provided with an individualized code. Once linked with a given user’s app, this code allowed researchers to track the user’s engagement with the Renew app through the secure research dashboard, including time-stamped button presses, time spent on each screen, number of characters typed into free text boxes, log-ins, and dates of use.

Consistent with previous mHealth app studies that have used multiple objective engagement criteria [15], we examined several indices of overall app engagement: total time spent in Renew, number of log-in days, and number of points gained in Renew. To specifically capture engagement with exposure, we examined time spent completing exposure activities (both imaginal and in vivo), the number of in vivo exposure activities completed, and the number of characters written during imaginal exposure. Number of characters was chosen as a proxy for the degree to which users engaged with their worst traumatic memory.

#### Posttraumatic Stress Symptoms

The PCL-5 [15] is a 20-item self-report measure of PTSD symptoms as defined by the *Diagnostic and Statistical Manual of Mental Disorders, Fifth Edition* (*DSM-5*) [16]. The PCL-5 has demonstrated strong psychometric properties [17,18] and has been validated for online use [19]. The Cronbach *α* in the current sample was 0.84. The PCL-5 was administered to each participant at 3 time points. For participants immediately assigned to one of the active use conditions, the PCL-5 was completed through REDCap before app use as part of the study screening procedures, after the 6-week active use period, and after a 6-week follow-up period. Participants assigned to the delayed use condition completed the PCL-5 before the delayed use period, at the end of the delayed use period before receiving a code to access Renew, and after their 6-week active use period.

### Procedures

The Stanford University Institutional Review Board approved the study. Informed consent was collected via telephone call, in which the study staff read a script that included information about Renew and the 3 study conditions to which they could be randomized. Study staff then allowed participants to ask questions about the study before providing consent. Eligible participants who consented to the study were randomly assigned to either 6 weeks of (1) Renew without study staff support, (2) Renew with a study support person as a support team member, or (3) delayed use. Neither participants nor study staff were blinded to conditions. Participants in the delayed use group were invited to use the app for 6 weeks after a 6-week wait period. Immediately before beginning the 6-week active use period, participants received a 30-minute app orientation by phone. During the orientation, a study staff member walked participants through each section of the app, described the features of Renew, and offered participants the chance to ask questions. Following the orientation, participants were then asked to use the app as often as they wanted for the next 6 weeks. Participants could choose to set reminders to use the app. Participants completed postuse measures through the REDCap online survey platform [13] at the end of the app-use period. Usage data were collected using a secure research dashboard. All participants who were assigned to the delayed use condition were given an access code for Renew at the end of the 6-week delayed use period. Data about their app usage was collected during a 6-week follow-up period, in addition to pre- and postuse symptom measures. For the current analysis, data were pooled from participants assigned to all 3 conditions, because there were no significant (*P*=.31) differences in PTSD symptom change during the active use periods across groups [9]. The majority of data collection took place online or through the online research dashboard that recorded Renew usage data, however, participants did complete postuse feedback interviews with study staff via telephone. Of the 93 participants who enrolled in the parent pilot trial [9], 69 participants had completed both pre- and postuse symptom measures and were included in this study.

### Data Analysis

Analyses were conducted using SPSS (version 26; IBM Corp) [20]. Descriptive and correlational analyses were conducted for all engagement variables. A hierarchical linear regression analysis was conducted with the overall app engagement variables entered in step 1 and exposure engagement variables entered in step 2. The dependent variable was change on the PCL-5. All app use data in the analysis came from the participants’ active use period (ie, for participants initially assigned to delayed use, usage data was taken from the active use period that followed the delayed use period).

## Results

Participant demographics are reported in Table 1. Participants were predominantly middle-aged, women, and White. More than half of participants in this subsample were army veterans (n=36, 52.17%), and the average PCL-5 score was well above the cutoff for clinically significant PTSD symptoms.

Means and SDs for user engagement variables are presented in Table 2.

**Table 1.** Participant demographic characteristics (N=69).

Variable	Value		
Age (years), mean (SD)	45.28 (9.05)		
**Gender** ^a^ **, n (%)**			
Men	22 (33.33)		
Women	47 (66.67)		
**Race or ethnicity** ^b^ **, n (%)**			
White	47 (68.12%)		
Black or African American	13 (18.84)		
Asian or Asian American	2 (2.89)		
American Indian or Alaska Native	4 (5.8)		
Middle Eastern or North African	1 (14.49)		
Other	2 (2.89)		
**Military branch, n (%)**			
Army	36 (52.17)		
Navy	13 (18.84)		
Air force	15 (21.74)		
Marines	5 (7.25)		
Baseline PCL-5^c^ score, mean (SD)	55.94 (9.87)		

a No participants identified as transgender, nonbinary, or another gender identity.

b Participants could select more than one race or ethnicity. Because of this, percentages sum to >100%.

c PCL-5: Posttraumatic Stress Disorder Checklist for *DSM-5*.

**Table 2.** Descriptive statistics for app engagement indices.

Overall engagement variables	Value, mean (SD)	
Log-in days	14.7 (10.71)	
Minutes in Renew	166.09 (156.52)	
Number of points gained	350.14 (451.42)	
**Exposure engagement variables**		
Number of in vivo exposure activities completed	1.84 (4.22)	
Minutes spent completing exposure activities	78.19 (135.35)	
Number of characters written during imaginal exposure activities	5116 (8522.43)	

Over the 6-week active use period, participants logged into Renew about one-third of the days. Users spent a little over 2.5 hours total engaging with Renew, not including their initial 30-minute orientation. Users spent over an hour engaging in exposure activities, averaging more than one complete in vivo exposure activity. In response to exposure prompts, participants wrote over 5000 characters on average, which roughly equals 750‐1000 words.

Correlations between app usage variables are presented in Table 3. Given the overlap in the engagement variables, there was high intercorrelation between the general and exposure-specific usage variables. Relationships between all variables were significant. The variable “minutes spent completing exposure activities” reflects minutes spent practicing both imaginal and in vivo exposure. Time spent on imaginal and in vivo exposure were combined in order to examine the impact of overall engagement with exposure in addition to the individual effects of engagement with imaginal and in vivo alone.

**Table 3.** Correlations (Pearson *r*) among usage variables.

Variables	Log-in days	Minutes in Renew	Number of points gained	Number of in vivo exposure activities completed	Minutes spent completing exposure activities	Number of characters written during imaginal exposure
**Overall usage variables**						
**Log-in days**						
*r*	1	0.74	0.81	0.47	0.41	0.45
*P* value	—^a^	<.001	<.001	<.001	<.001	<.001
**Minutes in Renew**						
*r*	0.74	1	0.79	0.64	0.65	0.54
*P* value	<.001	—	<.001	<.001	<.001	<.001
**Number of points gained**						
*r*	0.81	0.79	1	0.58	0.41	0.46
*P* value	<.001	<.001	—	<.001	<.001	<.001
**Exposure engagement variables**						
**Number of in vivo exposure activities completed**						
*r*	0.47	0.64	0.58	1	0.58	0.33
*P* value	<.001	<.001	<.001	—	<.001	.01
**Minutes spent completing exposure activities**						
*r*	0.41	0.65	0.41	0.58	1	0.38
*P* value	<.001	<.001	<.001	<.001	—	0
**Number of characters written during imaginal exposure**						
*r*	0.45	0.54	0.46	0.33	0.38	1
*P* value	<.001	<.001	<.001	.01	0	—

a Not applicable.

The results of the regression analysis are presented in Table 4. The initial model with overall engagement predicting PTSD symptom change was not significant and accounted for 1% of the variance observed in the model. However, the model with the exposure engagement variables added was significant and accounted for an additional 18% of variance in the model. Time spent completing exposure activities (*P*=.03) and the number of characters written during imaginal exposure (*P*=.009) were significant contributors to the final model.

**Table 4.** Hierarchical linear regression model (N=69).

Model steps and variables		β^a^	R	*R* ^2^	Δ*R*^2^	*F* test (*df*)	*P* value^b^	Δ*F* test (*df*)	*P* value^c^
**Step 1: overall engagement**		—^d^	0.1	0.01	—	0.18 (3,65)	0.9	—	—
	Log-in days	0.01	—	—	—	—	0.97	—	—
	Time in Renew	0.01	—	—	—	—	0.64	—	—
	Number of points gained	−0.02	—	—	—	—	0.53	—	—
**Step 2: exposure engagement**		—	0.44	0.19	0.18	2.31 (6,62)	0.04	4.42 (3,62)	0.01
	Number of in vivo exposure activities completed	−0.03	—	—	—	—	0.86	—	—
	Time spent completing exposure activities	0.36	—	—	—	—	0.03	—	—
	Number of characters written during imaginal exposure	0.37	—	—	—	—	0.01	—	—

a β: Standardized beta weight.

b Significance for standardized beta weights of predictors and steps of the model (*F* test statistic).

c Significance for change in *F* test statistic (Δ*F* test) at second step.

d Not applicable.

## Discussion

### Overview

We examined the relationship between engagement with imaginal and in vivo exposure and PTSD symptom reduction in an mHealth app, Renew. Few randomized controlled trials have examined mobile app-delivered interventions for PTSD [21], and even fewer studies have evaluated the impact of usage factors on symptom change in digital PTSD interventions [22,23]. As hypothesized, we found that engagement with exposure was associated with greater PTSD symptom reduction over and above overall use of the app in general. Our finding that overall app use was unrelated to PTSD symptom change is consistent with previous research on PTSD Coach [6] but inconsistent with meta-analytic findings showing that engagement with mHealth apps in general is associated with symptom change [24]. To our knowledge, this is the first study to demonstrate a relationship between engagement with the active therapeutic components of an mHealth app and improvement in PTSD symptoms.

Our findings are consistent with the limited research on the relationship between app usage and symptom change in self-management apps for depression and anxiety, which suggests that higher rates of engagement with active intervention components are associated with symptom change. Specifically, Zhang et al [25] found that participants’ depressive symptom reduction was associated with greater engagement in self-tracking and moderate engagement with learning and goal setting, but anxiety symptom reduction was unrelated to engagement with specific app components. One other study, Nardi et al [26], examined data from a clinical trial of a mindfulness app and also found evidence that engagement with specific components, such as meditation, was linked to improvement in generalized anxiety symptom reduction. To our knowledge, there are no studies of PTSD apps that have examined how engagement with different intervention components relates to symptom change. Even among apps for more common mental health concerns, robust reporting on usage metrics remains scant [27]. Our analysis of exposure-specific use metrics in this study contributes to a profound gap in the literature on digital mental health interventions. Our finding that exposure may be helpful for reducing PTSD symptoms through a self-guided mHealth app is consistent with the broader literature on efficacious PTSD interventions [28]. Future work should examine digital interventions that include active cognitive behavioral therapy (CBT) components such as exposure, which are associated with symptom change. Indeed, in a large meta-analysis of 176 randomized controlled trials, effect sizes were larger for self-management apps for anxiety and depression that included CBT components compared to those that did not [29].

The number of characters entered during imaginal exposure, which we considered a proxy for the degree to which participants engaged with and recounted their worst traumatic memory, and the time spent in any exposure activity, both significantly contributed to the model predicting PTSD symptom change, whereas the number of exposure activities did not. This may suggest that the depth of engagement and time spent approaching trauma-related memories and situations is important for therapeutic change in the context of mHealth apps for PTSD. This pattern of findings maps to the literature on in-person psychotherapy, where most exposure therapies emphasize the importance of imaginal exposure [30].

### Limitations

Several study limitations should be noted. Data for this study were drawn from a pilot trial, and replication of the current findings is needed. Participants were veterans with clinically significant symptoms of PTSD; findings may not generalize to other populations diagnosed with PTSD. Data were cross-sectional, which prevents causal interpretations. In addition, many of the usage variables were highly intercorrelated and there is no established gold standard approach for operationalizing app engagement. These limitations notwithstanding, this study provides preliminary evidence of a unique relationship between engagement with exposure and PTSD symptom change among veterans with clinically significant symptoms of PTSD who used a self-guided mHealth app (Renew) for a 6-week period.

## Abbreviations

*DSM-5*: *Diagnostic and Statistical Manual of Mental Disorders, Fifth Edition*
mHealth: mobile health
PCL-5: Posttraumatic Stress Disorder Checklist for *DSM-5*
PTSD: posttraumatic stress disorder
RedCap: Research Electronic Data Capture
VA: Department of Veterans Affairs
